# The complete plastome sequence of *Artocarpus altilis* (Parkinson ex F.A. Zorn) Fosberg, (Moraceae)

**DOI:** 10.1080/23802359.2022.2163595

**Published:** 2023-01-16

**Authors:** Lv Wei, Hong-Xin Wang, Da-Juan Chen, Xiu-Rong Ke, Zhi-Xin Zhu, Hua-Feng Wang

**Affiliations:** a College of Tropical Crops, Hainan Key Laboratory for Sustainable Utilization of Tropical Bioresources, Hainan University, Haikou, China; b Zhai Mingguo Academician Work Station, Sanya University, Sanya, China; c Sanya Nanfan Research Institute of Hainan University, Sanya, Hainan, China

**Keywords:** *Artocarpus communis*, Moraceae, plastome, genome structure

## Abstract

*Artocarpus altilis* (Parkinson ex F.A. Zorn) Fosberg is native to the Pacific Islands, India, and the Philippines. It is also cultivated in Taiwan and Hainan. The complete plastome of the species was assembled and annotated in this study. The circular genome was 160,184 bp in size, presenting a typical quadripartite structure including two inverted repeats (IRs) of 25,734 bp, a large single-copy (LSC) of 88,791 bp, and a small single-copy (SSC) of 19,925 bp. The genome contained 132 genes, including 87 protein-coding genes, 37 tRNA genes, and eight rRNA genes. The total G/C content of complete plastome was 36.0%, with the corresponding values of the LSC, SSC, and IR being 33.7%, 28.8%, and 42.7%, respectively. The complete plastome sequence of *A. altilis* (Parkinson ex F.A. Zorn) Fosberg will make contributions to the conservation genetics of this species as well as to phylogenetic studies of Moraceae.

*Artocarpus altilis* (Parkinson ex F.A. Zorn) Fosberg 1941 is an important plant species in the family Moraceae, which is native to the Pacific Islands, India, and the Philippines. It is one of the more famous tropical trees in the Malay Islands. *A. altilis* (Parkinson ex F.A. Zorn) Fosberg has high economic value (Lathiff et al. 2021) and is famous for its edible fruits. It is also cultivated in Hainan and Taiwan of China. Recently, rapid progress has been made in the family Moraceae genome sequencing and analysis, but because of the complexity and order of magnitude increase in genome sizes, similar progress has not been attained for *A. altilis* (Parkinson ex F.A. Zorn). Furthermore, it is the staple food in many tropical regions, and is one of the most productive edible plants. The wood can be used in light buildings, crates, large canoes, and boats. The bark is very thick and tough for ropes or weave cloth. The leaves are edible or used as medicine of reducing fever. Here, we report the complete plastome of *A. altilis* (Parkinson ex F.A. Zorn) Fosberg to improve the quality of collections and phylogenetic studies of Moraceae.

Fresh leaves were collected in Qionghai, Hainan (97.84°E, 24.00°N) and stored in silica gel. The specimen (certificate code: D.-J. Chen, X.-R. Ke, A88, HUTB) and associated DNA are stored in the Herbarium of the National Gene Bank of China (code of herbarium: HUTB, http://sweetgum.nybg.org/science/ih/herbarium-details/?irn=124686, contact for the Specimen and DNA, H.-F., Wang, hfwang@hainanu.edu.cn).

The experiment was carried out as reported in Zhu et al. (2018). We constructed paired-end sequencing libraries with insert sizes of 300–500 bp with Illumina TruSeq™ Nano DNA Sample Prep Kit and sequenced using the BGISEQ-500 at the Beijing Genomics Institution (BGI; Shenzhen, China). Clean sequencing data were assembled with GetOrganelle v1.7.5.0 (Jin et al. 2020). The assembled plastome was annotated against the published plastome of *Artocarpus camansi* Blanco 1837 (NC_054247.1) by using Geneious Prime v2021.2.2 (Biomatters Ltd., Auckland, New Zealand) and the annotation was corrected with DOGMA (Wyman et al. 2004).

Our results show that the complete plastome has a full length of 160,184 bp with a typical quadripartite structure of angiosperms consisting of 25,734 bp for the two inverted repeats (IRs), 88,791 bp for the large single-copy (LSC) region, and 19,925 bp for the small single-copy (SSC) region. The plastome consists of 132 genes, consisting of 87 protein-coded genes copied (six of which are duplicated in the IR), 37 tRNA genes (seven of which are duplicated in the IR), and eight rRNA genes (5s rRNA, 4.5s rRNA, 23s rRNA, and 16s rRNA) (four of which are duplicated in the IR). The total G/C content of the plastome is 36.0%, and the G/C content of the LSC, SSC, and IR regions is 33.7%, 28.8%, and 42.7%, respectively.

The best performing model of molecular evolution was selected as the GTR + I+G model by MrModelTest v2.4 (Posada 2008). We inferred the chloroplast genome phylogenetic relationships with CIPRES (http://www.phylo.org/portal2/login!input.action) based on existing data of related taxa from NCBI. We find that *A. altilis* (Parkinson ex F.A. Zorn) Fosberg is closer to *A. camansi* Blanco 1837 than other species of Moraceae included in this study (Figure 1). The majority of nodes in the plastome ML tree shows strong support. The plastid sequence of *A. altilis* (Parkinson ex F.A. Zorn) Fosberg will promote relevant conservation and phylogenetic investigations of Moraceae, bringing great benefits to deepen the understanding of phylogenies within Moraceae.

**Figure 1.**
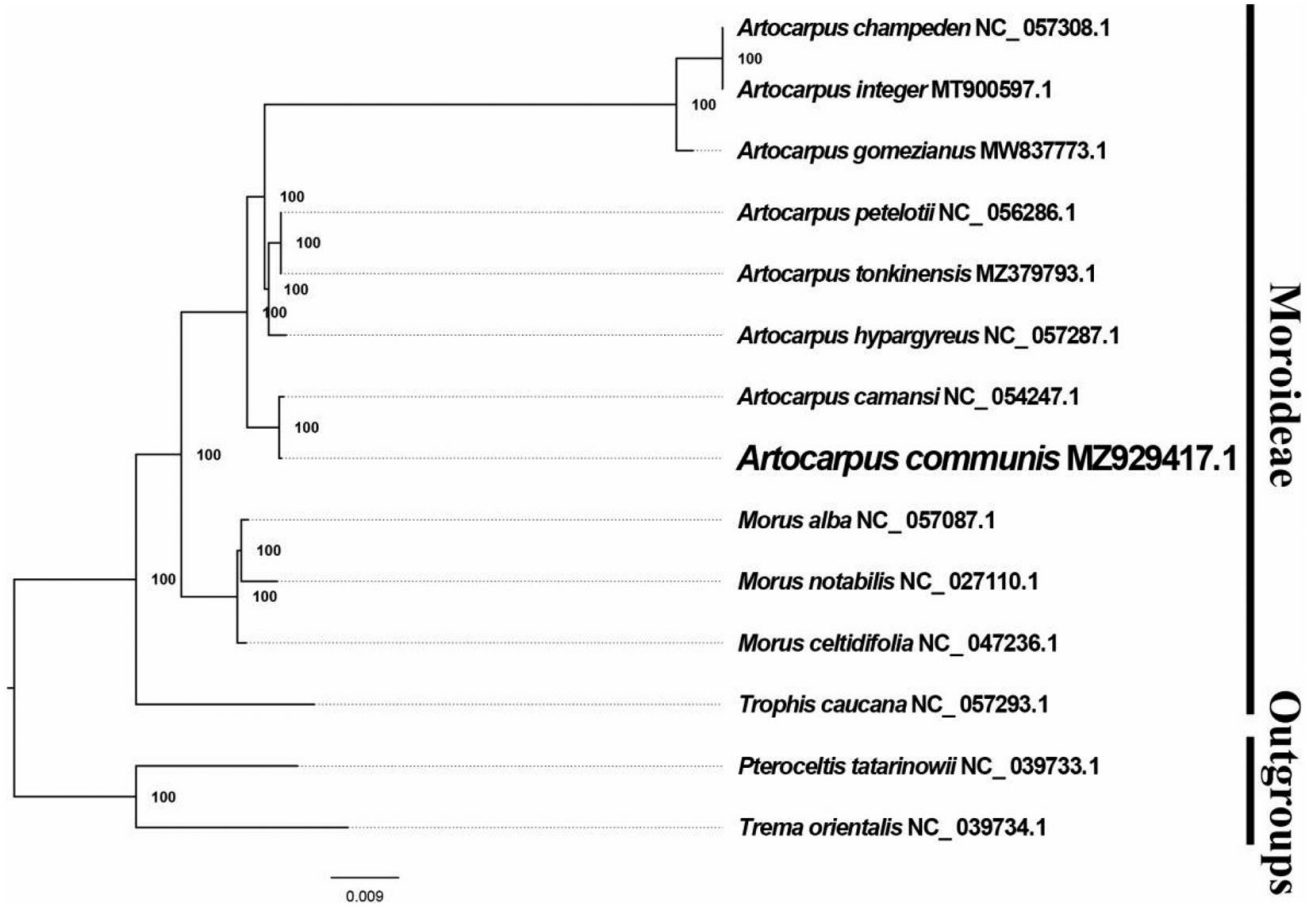
The best ML phylogeny recovered from 14 complete plastome sequences using RAxML.

## Data Availability

The genome sequence data supporting the results of this study are publicly available in GenBank of NCBI (https://www.ncbi.nlm.nih.gov/) with registration number MZ929417.1. The associated BioProject, SRA, and Bio-Sample numbers are PRJNA748537, SRR15651127, and SAMN20858446, respectively.
